# Addressing the Needs of Migrant Workers in ICUs in Singapore

**DOI:** 10.1177/2382120520977190

**Published:** 2020-12-18

**Authors:** Crystal Lim, Jamie Xuelian Zhou, Natalie Liling Woong, Min Chiam, Lalit Kumar Radha Krishna

**Affiliations:** 1 Medical Social Services, Singapore General Hospital, Singapore; 2 Division of Supportive and Palliative Care, National Cancer Centre Singapore, Singapore; 3 Academic Clinical Programme, SingHealth Duke-NUS Academic Medical Centre, Singapore; 4 Lien Center for Palliative Care, Duke-NUS Graduate Medical School, Singapore; 5 Department of Internal Medicine, Singapore General Hospital, Singapore; 6 Division of Cancer Education, National Cancer Centre Singapore, Singapore; 7 Palliative Care Institute Liverpool, Academic Palliative & End of Life Care Centre, Cancer Research Centre, University of Liverpool, UK; 8 Centre for Biomedical Ethics, National University of Singapore, Singapore; 9 PalC, The Palliative Care Centre for Excellence in Research and Education, Singapore

**Keywords:** Intensive care units, translations, emigrants and immigrants, training support, medical interpretation, medical translation, migrant community, COVID-19

## Abstract

**Background:**

With nearly 400 000 migrant workers in Singapore, many from Bangladesh, India and Myanmar, language and cultural barriers posed a great many challenges during the COVID-19 pandemic. This was especially so as majority of the COVID-19 clusters in Singapore emerged from their communal dormitories. With concerns arising as to how this minority group could be best cared for in the intensive care units, the need for medical interpreters became clear.

**Main:**

In response, the Communication and Supportive Care (CSC) workgroup at the Singapore General Hospital developed the ‘Medical Interpreters Training for ICU Conversations’ program. Led by a medical social worker-cum-ethicist and 2 palliative care physicians, twenty volunteers underwent training. The program comprised of 4 parts. Firstly, volunteers were provided with an overview of challenges within the COVID-19 isolation ICU environment. Discussed in detail were common issues between patients and families, forms of distress faced by healthcare workers, family communication modality protocols, and the sociocultural demographics of Singapore’s migrant worker population. Secondly, key practice principles and ‘Do’s/Don’ts’ in line with the ethical principles of medical interpretation identified by the California Healthcare Interpreters Association were shared. Thirdly, practical steps to consider before, during and at the end of each interpretation session were foregrounded. Lastly, a focus group discussion on the complexities of ICU cases and their attending issues was conducted. Targeted support was further provided in response to participant feedback and specific issues raised.

**Conclusion:**

As a testament to its efficacy, the program has since been extended to the general wards and the Ministry of Health in Singapore has further commissioned similar programs in various hospitals. In-depth training on the fundamentals of medical terminology, language and cultural competency should be provided to all pertinent healthcare workers and hospitals should consider hiring medical interpreters in permanent positions.

## Introduction

Singapore boasts of a multicultural society with 4 official languages – Malay, Tamil, various dialects of Chinese and English, its main language of instruction.^1^ However of its 5.7 million population, many Singaporeans do not speak Bengali, Hindi, Kannada, Malayali, Telegu and Burmese, the dominant languages of the 400 000 migrant workers in Singapore, many from Bangladesh, India and Myanmar.^2^

This communication gap became a significant issue on 30th March 2020 when the first COVID-19 cluster amongst migrant workers was detected in the communal living dormitories. By 31st May 2020, more than 32 000 were tested positive.^3^ Whilst most were deemed healthy and relocated to purpose-built community isolation facilities, concerns arose as to how they could be best cared for in intensive care units (ICU)s amidst growing data that 5% to 8% of COVID-19 cases would require ICU admissions.^4^

This quandary raised unique communication challenges. Traditionally, family members provided emotional and spiritual support as well as informal translations for patients. However to curb the spread of infection, restrictions placed on travel and hospital visitations saw the obfuscation of this vital source. There was a clear need for medical interpreters who could effectively liaise between the medical professionals and families of these migrant workers, many of whom were residing in their own native countries. To adequately prepare these volunteer interpreters, the Communication and Supportive Care (CSC) workgroup at the Singapore General Hospital developed a 4-part ‘Medical Interpreters Training for ICU Conversations’ program.

## ‘Medical Interpreters Training for ICU Conversations’ Program

Twenty doctors, nurses and research fellows were identified as potential interpreters due to their language expertise and medical background. With a firm grasp of medical terminologies and clinical procedures, they were perceived to be better prepared for the complex care of ICU patients. All 20 generously agreed to volunteer.

Well-versed in medical education and communication skills training, the program was spearheaded by a medical social worker-cum-ethicist and 2 palliative care physicians who worked in the isolation wards. The session was conducted over Zoom and lasted 1.5 hours with 2 time slots made available to allow for a smaller trainer-participant ratio. As pre-course preparation, a list of common ICU terms was given to the volunteers to practise interpreting into layman vocabulary.

### Context of COVID-19 within the isolation ICUs

In the first segment, the trainers provided the volunteers with a snapshot of challenges within the isolation ICU environment. Discussed in detail were communication and interrelational issues between patients and families and the emotional and moral distress faced by healthcare workers. The hospital’s family communication modality protocols were also carefully delineated with online etiquette regarding the use of Zoom and WhatsApp to provide medical updates and opportunities for final farewells clearly conveyed.

Further elucidated were the sociocultural demographics of the migrant worker population in Singapore. Areas highlighted were their relatively low socio-economic status and health literacy as well as their lack of familiarity with government policies regarding COVID-19 treatment (eg, free treatment for infected migrant workers). Coming from rural areas or locations under-served by their local health systems saw their limited understanding and resistance towards Western medicine and intensive care. Whilst some displayed a lack of trust towards authority, others displayed a tendency towards complete deference, diminishing their own regard for autonomous decision making.

### Practice principles of medical interpretation

In the next segment, key practice principles were shared (Table 1). Aligning with the ethical principles of interpretation identified by the California Healthcare Interpreters Association (CHIA), the importance of ‘confidentiality, impartiality, respect of individuals and their communities, professionalism and integrity, accuracy and completeness and cultural responsiveness’ was foregrounded.^5^

**Table 1.** Key practice principles of medical interpretation.

Do’s	Don’ts
Use ‘first person’ reference (eg, I, you).	Use third person reference (eg, ‘The doctor says’, ‘The family says’, ‘They say’).
Remind communicating parties to speak in small chunks.	Have side conversations with either party.
Establish a direct relationship between the doctor and the family.	Allow either party to speak through you, hence becoming a messenger instead of an interpreter.
Explain medical terms in layman language (eg, a breathing tube is placed into the patient’s mouth).	Use medical jargon (eg, intubation).
Interpret based on meaning rather than word-for-word.	Change or omit content of source language.
Maintain neutrality in interpretation.	Take sides.
Ensure completeness and accuracy when interpreting.	Vary or alter communication content due to own discomfort.
Seek clarification if unsure of meaning of a term used by any party.	Allow personal opinion or feelings to alter the interpretation.
Ask doctor for follow-up action, if any.	Carry out other tasks other than interpretation (eg, help the doctor contact an embassy).
Maintain confidentiality.	
Observe one’s own pace and tone of speech.	

### Practical steps before, during and at the end of the interpretation session

Practical steps before, during and at the end of the interpretation session were also relayed to the volunteers. These guidelines comprise of the following:

Before the session, the patient’s prevailing condition and treatment plan should be clearly communicated to the interpreter by the medical team. The family should be informed of the patient’s hospitalisation at the earliest possible and the doctor’s intent for a family conference should be made known. A suitable time should be arranged and the feasibility of a phone or video call discussed. The family should be pre-empted that an interpreter will be present to assist with communications and basic interpretation principles must be relayed to the medical team in advance to facilitate the session (eg, giving the interpreter permission to pause the conversation).

During the session, the family should be primed of the role of the medical interpreter.^5^ Physician volunteers must explicitly state that they are serving solely as interpreters and are not involved in the medical team’s decision making. If the interpreter wishes to share their observations, they should be clearly prefaced with the line, ‘As the interpreter, I am sensing that. . .’ or its equivalent. Just as it is important to inform the medical team if the family requires clarification, they must also identify any tension or misunderstandings. Any cultural barriers or nuances observed should also be swiftly raised.

At the end of the session, a clear summary and subsequent follow-up actions must be reiterated to ensure that the patient, their family and the medical team are on the same page. This will minimise confusion and reduce the risk of blame being placed on the interpreter for mistranslations or omission of information.^6,7^

### Case study

Finally, to immerse the volunteers in plausible scenarios, a case study was presented to convey the complexities of ICU cases and their attending issues. This focus group discussion enabled the volunteers to better appreciate (i) the context in which interpretation may take place, (ii) the content of communications in the ICU and (iii) the emotional and psychological distress that patients and their families may wrestle with.

## Participants’ Feedback

The participants provided rich feedback and 3 important points were highlighted.

1. Despite their medical background, some of the volunteers reported personal discomfort with decisions surrounding treatment withdrawal. They further expressed that this concept would be alien to the layman.^8^ As such, a session on Singapore’s ethical and legal standpoint on the withdrawal of life-sustaining treatments was incorporated into the program.^9-10^
2. As experienced clinicians, some found that taking on the role of a neutral and passive interpreter proved difficult. Whilst the trainers encouraged them to retain their neutrality and objectivity, they were advised to clarify with the medical team if any breaches were suspected.
3. The volunteers also voiced the importance of a safe space to air their concerns. As a result, debriefs were conducted after every interpretation session to help the volunteers navigate through their experience and come to terms with the extraordinary circumstances they faced in the ICU.

## Conclusion

The COVID-19 surge within the migrant worker community saw a plethora of challenges as a result of steep language barriers, low health literacy rates and limited access to loved ones halfway across the globe. The Singapore General Hospital’s medical interpretation training program sought to streamline their healthcare delivery and alleviate their attending concerns. As a testament to its efficacy, the program has since been extended to the general wards, with positive feedback that the training effectively armed them with practical knowledge and skills. Indeed, the Ministry of Health in Singapore has further commissioned similar programs in various hospitals. With clear benefits reaped and to upscale beyond the pandemic, in-depth training on the fundamentals of medical terminology, language and cultural competency should be provided to all pertinent healthcare workers. In addition, hospitals should also consider hiring medical interpreters in permanent positions as they have shown to be valuable assets in the streamlining of doctor-patient communications and the delivery of compassionate care.

## References

[1] Languages of Singapore. En.wikipedia.org. Published 2020. Accessed September 23, 2020 https://en.wikipedia.org/wiki/Languages_of_Singapore

[2] CNA. The big read: solving Singapore’s foreign workers problem requires serious soul searching, from top to bottom. Published 2020. Accessed September 23, 2020 https://www.channelnewsasia.com/news/singapore/coronavirus-covid-19-foreign-workers-big-read-dormitories-12718880

[3] MOH. Updates on COVID-19 (Coronavirus Disease 2019) Local Situation. Moh.gov.sg. Published 2020. Accessed September 23, 2020 https://www.moh.gov.sg/covid-19

[4] Maves RC Downar J Dichter JR, et al Triage of scarce critical care resources in COVID-19 an implementation guide for regional allocation: an expert panel report of the task force for mass critical care and the American college of chest physicians. Chest. 2020;158:212-225. 32289312 10.1016/j.chest.2020.03.063 PMC7151463

[5] Angelelli C Agger-Gupta N Green C Okahara L The California standards for healthcare interpreters: ethical principles, protocols and guidance on roles and intervention. In: Wadensjö C Dimitrova B Nilsson A-L, eds. The Critical Link 4: The Professionalization of Interpreting in the Community. John Benjamins Publishing Company; 2007:167-180.

[6] Hsieh E Kramer E. Medical interpreters as tools: dangers and challenges in the utilitarian approach to interpreters’ roles and functions. Patient Educ Couns. 2012;89:158-162. 22857777 10.1016/j.pec.2012.07.001 PMC3462307

[7] Pham K Thornton J Engelberg R Jackson J Curtis J. Alterations during medical interpretation of ICU family conferences that interfere with or enhance communication. Chest. 2008;134:109-116. 18347204 10.1378/chest.07-2852 PMC2693085

[8] Phua J Joynt G Nishimura M Deng Y Myatra S Chan Y, et al Withholding and withdrawal of life-sustaining treatments in intensive care units in Asia. JAMA Intern Med. 2015;175:363. 25581712 10.1001/jamainternmed.2014.7386

[9] Mental Capacity Act - Singapore Statutes Online. Sso.agc.gov.sg. Published 2020. Accessed September 23, 2020 https://sso.agc.gov.sg/Act/MCA2008

[10] Advance Medical Directive Act - Singapore Statutes Online. Sso.agc.gov.sg. Published 2020. Accessed September 23, 2020 https://sso.agc.gov.sg/Act/AMDA1996

